# Editorial: Transitional Care Clinics to Reduce 30-day Readmissions in Heart Failure Patients

**DOI:** 10.7759/cureus.2069

**Published:** 2018-01-15

**Authors:** Katherine Smith, Jeffrey P Fleming, Bernard Gros

**Affiliations:** 1 Medical Education, UCF College of Medicine; 2 Internal Medicine, University of Central Florida

**Keywords:** heart failure, affordable care act, hrrp, transitional care, readmissions, quality metrics, interdisciplinary care

## Abstract

Heart failure (HF) is one of the most prevalent chronic diseases in the United States (U.S.), with an estimated prevalence of five million patients in the U.S. and an annual healthcare cost of greater than 30 billion dollars. Readmission rates for HF patients are astronomically high, with up to 25% of hospitalized patients with HF requiring readmission within 30 days of discharge. The Hospital Readmissions Reduction Program (HRRP) of the Patient Protection and Affordable Care Act aims to address these concerns by financially penalizing institutions with unacceptably high risk-adjusted 30-day readmission rates for HF patients. The introduction of the HRRP contributed to increased efforts of healthcare systems to reduce their 30-day readmission rates, often by the utilization of “transitional care clinics.” Although the motivation for the creation of these programs is understandable, there exists a paucity of robust clinical trials supporting the efficacy of these programs to reduce 30-day readmission rates for HF patients. There is even less evidence to support the use of these programs in the unique healthcare environment of the U.S. Large, multicenter randomized controlled trials should be conducted to evaluate these interventions before more resources are dispersed toward their implementation. Alternatively, resources could be used to evaluate other interventions that may be more efficacious at reducing 30-day readmissions, such as implantable hemodynamic monitoring devices.

## Editorial

Heart failure (HF) represents a significant clinical and public health burden in the United States (U.S.), with a prevalence of five million patients in the U.S. [1]. This condition causes great strain to the economic resources of our healthcare system, with HF expenditures estimated at 31 billion dollars in the U.S. in 2012 [2]. Much of this expenditure is toward hospital readmissions, as approximately 25% of index HF admissions will be followed by a second admission [1]. These challenges were a driving force toward the inception of the Hospital Readmissions Reduction Program (HRRP) of the Patient Protection and Affordable Care Act. Under this legislation, institutions with unacceptably high 30-day readmission rates for several conditions, including HF, are faced with financial penalties [3]. The introduction of the HRRP created an interesting scenario for healthcare institutions in the U.S. Previously, a reduction in total healthcare expenditures resulting from lower readmission rates would have likely reduced income for healthcare institutions. Now, interventions that reduce readmission rates for HF can be cost-effective to the patient while aligning with the overall financial interest of the healthcare system in which it is instated.

The HRRP was the driving force for the creation of many “transitional care programs” for post-discharge HF patients in the U.S. These programs aim to reduce HF readmission rates by targeting known precipitants of readmission. These include poor transitions of care, lack of access to post-discharge care, inadequate access to medications, medication non-compliance, and improper diet with excess salt intake [4].They employ a variety of techniques, including telemonitoring, structured telephone support, home healthcare services, patient education, and interdisciplinary outpatient clinics to improve patient well-being. Interdisciplinary care clinics are the most comprehensive and intensive outpatient programs provided to post-discharge HF patients. Most of these programs provide primary care, specialist follow-up, and comprehensive patient education with coaching to promote self-management including medication and dietary compliance. Transitional care clinics utilize providers from multiple disciplines, including medicine, nursing, pharmacy, social work, and nutrition. Ideally, these programs should prevent the duplication of services, medication confusion, and patient anxiety.

The drive for implementation of these clinics is understandable, but caution is warranted as the evidence supporting the efficacy of these programs is weak. A review of transitional HF clinics published by Feltner, et al. in 2014 concluded that these multidisciplinary HF clinics and home-visiting programs reduced all-cause readmission and mortality in HF patients [4]. Their analysis also found that structured telephone support programs reduced HF-specific readmission and mortality. Although these results suggest a possible benefit with the implementation of these programs, their evaluation was limited by the studies included in their analysis. Much of the published research on this topic provides low-quality evidence, with most data sourced from studies with severe bias, lack of proper controls, and inconsistent reporting of outcomes. Furthermore, many of the studies evaluating these interventions were conducted outside of the U.S. It is unclear whether any reported success of these interventions can translate to our unique healthcare system. Ultimately, in the era of evidence-based medicine, the jury is still out on the utility of these transitional heart failure programs.

Although there is weak evidence supporting transitional care clinics and other outpatient readmission reduction programs, there are promising results for the implementation of remote monitoring of pulmonary artery pressures with wireless implantable devices. Such devices were evaluated by a large, multicenter single-blind clinical trial entitled The CardioMicroelectromechanical system (CardioMEMS Inc., Atlanta, Georgia) Heart Sensor Allows Monitoring of Pressures to Improve Outcomes in New York Heart Association Class III Heart Failure Patients (CHAMPION) Trial (ClinicalTrials.gov Identifier: NCT00531661) [5]. The CHAMPION trial revealed a 58% reduction in 30-day readmission rates in the group implanted with the remote monitoring device (hazard ratio 0.42, 95% confidence interval 0.22–0.80; P=0.0080). These results offer a promising tool for the post-discharge management of HF patients where transitional care clinics were unable to provide robust data.

With the inception of the HRRP, there has been significant focus on quality improvement initiatives and the reduction of 30-day readmission for HF patients. Although this policy provides understandable motivation to implement transitional care clinics in the U.S., the investment of significant resources into these programs should be questioned. The literature offers weak support for these programs as efficacious means to reduce 30-day readmissions for HF patients with few multicenter randomized controlled trials offering supportive evidence for these programs. Robust trials should be developed to scrutinize the use of transitional care clinics for the post-discharge management of HF patients. Specifically, these studies should be multicenter randomized control trials using demographically diverse populations who are medically optimized prior to discharge. Conclusions should be drawn in the context of the patient New York Heart Association classification and discharge ejection fraction to allow practitioners to make an evidence-based decisions when enrolling patients in a given intervention. Alternatively, funds should be distributed toward the development and implementation of evidence-based approaches to reduce 30-day readmission rates for HF patients, such as implantable hemodynamic monitors.
